# The effect of employees’ sense of power on supervisors’ voice endorsement: A cross-level moderated mediation model

**DOI:** 10.1371/journal.pone.0269427

**Published:** 2022-10-07

**Authors:** Ping Yuan, Yuan Cheng, Yanbin Liu, Fanghui Ju

**Affiliations:** 1 Logistics and E-commerce College, Zhejiang Wanli University, Ningbo, China; 2 School of Business, NingboTech University, Ningbo, China; Polytechnic Institute of Coimbra: Instituto Politecnico de Coimbra, PORTUGAL

## Abstract

Based on expectation states theory, we examined the mechanism underlying the effect of employees’ sense of power on supervisors’ voice endorsement, and tested our hypothesized model on a sample of 307 employees from 60 work teams. We used a two-time lagged design and paired questionnaire survey. Our analysis indicated that employees’ sense of power enhanced supervisors’ voice endorsement, and supervisors’ perceived voice constructiveness mediated this relationship. Multilevel analyses showed that power distance negatively moderated the influence of sense of power on perceived voice constructiveness and negatively moderated its indirect effect on voice endorsement.

## Introduction

With increasing environmental uncertainly in organizations, it becomes inefficient to rely solely on managers’ personal abilities to cope with organizational problems. To avoid organizational risks and improve workplace efficacy, it is important for organizations to encourage employees to express their work-related opinions and suggestions freely [1–3]. However, a precondition of voice behavior benefitting an organization is that employees’ voices are endorsed by supervisors. Researchers have investigated various antecedents of supervisors’ voice endorsement, such as supervisors’ power [4], advisors’ facial width-to-height ratio [5] and emotion [6], expertise and trustworthiness [7,8], the quality of leader–member exchange [9,10], and voice politeness and directness [11]. Although these studies have highlighted the key antecedents of voice endorsement, unanswered questions remain.

First, the relationship between the advisor’s personal power and voice endorsement needs to be further examined. Previous research explored the roles of voice content and advice receivers on voice endorsement [4,11], but the characteristics of advisors are also important. Supervisors will evaluate employees based on certain social cues. For example, employees with a greater sense of power have more agency [12]; thus, supervisors will evaluate the performance of such employees positively [13], which might lead to stronger voice endorsement. Second, the mechanism of the advisor’s sense of power on voice endorsement is worthy of investigation. Theoretical frames explaining the effect of sense of power on voice endorsement have focused on leader–member exchange [10] and advisors’ social influence [11]. However, expectation states theory [14,15] suggests that supervisors will expect employees with a greater sense of power to exhibit better performance; thus, the constructiveness of such employees’ voices is more easily identified by their supervisor. Research suggests that the ability of an advisor leads to voice endorsement [7], but a supervisor’s perception of an advisor’s ability is influenced by the advisor’s power [16]. Third, the situational factors influencing the impact of advisor’s power on voice endorsement needs to be discussed. Previous research has indicated that voice endorsement is a consequence of various factors and should be considered from a range of perspectives [9,17], so it is valuable to discuss the mechanism, as well as the situational factors in the relation between sense of power and voice endorsement.

This study is among the first in investigating the antecedent of supervisor’s voice endorsement from the perspective of employee’s personal psychological characteristic, i.e. their sense of power. We aimed to reveal the underlying mechanism and situational factors in the relation between an employee’s sense of power and his or her supervisor’s voice endorsement. We make three contributions to the literature. First, based on expectation states theory [15], we would enrich the theoretical research about the antecedents of voice endorsement [11]. Second, by introducing supervisors’ perceived voice constructiveness, we explain the underlying mechanism of the impact of employees’ sense of power on supervisors’ voice endorsement, which deepens the understanding of how sense of power leads to voice endorsement. Third, we developed a cross-level moderated mediation model to test our hypotheses that power distance negatively moderates the influence of employees’ sense of power on supervisors’ perceived voice constructiveness and negatively moderates its indirect effect on voice endorsement. Our findings extended the knowledge about the impact of employees’ characteristics on supervisors’ voice endorsement, also improved our understanding of how and when employees’ sense of power would influence the acceptance of their voice behavior, which would gain insights for management practice in organizations.

## Rationale for the study

Expectation status theory [14] demonstrated that in task groups, people develop a differentiated expectation of another’s performance guided by social cues, and further make an assessment about their contribution to the task at hand. People with more favorable expectations have greater likelihoods of better assessment [18]. According to this theory, gender, race, age and education level are among the key social cues from which supervisors and colleagues develop performance expectations and make assessments [18].

Research has shown that agency and communion are two fundamental dimensions on which perceptions of individuals and groups are based [19]. A high degree of agency is associated with better personal ability and higher confidence, while positive communion indicates more concern for the welfare of group members [20]. Individuals with a stronger sense of power behave more proactively, for instance expressing their personal opinions frequently and freely [13], which is a sign of strong personal ability [21]. Speaking up frequently may also make a supervisor feel that the advisor is other-orientated [21]. Thus, we assume that employees with a stronger sense of power will be evaluated by their supervisor as having better personal ability and being more communal and helpful for the organization, and their opinions will be more valuable for the organization, which can lead to stronger voice endorsement. Previous research has also shown that people with a greater sense of power will express their opinions more frequently and firmly, and thus be considered to have better personal ability and better communication [21,22]. Thus, we focus on expectation status theory to explain the effect of an employee’s sense of power on his or her supervisor’s voice endorsement.

### Sense of power and voice endorsement

Compared to individuals with a low sense of power, people with a strong sense of power focus more on the link between effort and reward, and express their personal opinions more often [23] and are more confident [13]. These characteristics might lead to more identification of their voice behavior in the workplace. In task groups, individuals with a high sense of power take others’ thoughts less seriously and will undertake more promotive strategies and behaviors to achieve their task performance. As they are more confident, employees with a high sense of power are more proactive and willing to take risks [13]. Employees with a high sense of power also dare to express and defend their opinions, which increases supervisors’ perception of their prosocial attitudes toward the welfare of the group [24]. These social cues indicating that employees with a high sense of power are “confident,” “willing to take risks,” and “proactive” will help to shape supervisors’ cognition about their contribution to the organization, for example, “This employee makes more effort in accomplishing tasks,” “This employee devotes more energy to achieving goals,” and “This employee contributes more to thinking about how to avoid risk and improve efficiency in the organization,” which will lead to a more positive expectation of the target employee’s performance. Thus, this employee’s suggestions will be more highly evaluated and more easily accepted by the supervisor. We therefore propose the following hypothesis:

H1: Employees’ sense of power has a positive influence on their voice endorsement.

### The mediating role of voice constructiveness

Employees with high sense of power would lead to stronger perceptions of their voice constructiveness. Expectation states theory suggests that individuals expressing positive social cues will have positive performance expectations and their behaviors will be endowed with more value [15]. Compared to people with a low sense of power, people with a high sense of power behave more proactively. Based on these behavioral cues, supervisors will assume that employees with a high sense of power make bigger contributions to accomplishing work tasks and will make more positive evaluations of them. Thus, when such employees engage in voice behavior, supervisors view their suggestions as constructive. In conclusion, employees’ sense of power positively influences supervisors’ judgments of their voice constructiveness. Previous research has also shown that individuals’ agency and communion lead to more positive evaluations [21,22].

Supervisors’ perceptions of voice constructiveness increase the possibility that employees’ voices will be endorsed. When an employee’s voice is believed to be constructive, that is, their suggestions are helpful for developing organizational performance and avoiding potential problems, supervisors will tend to endorse their suggestions. In contrast, when perceived voice constructiveness is low, their advice will not be believed to have a positive effect on the organization and so will be more likely to be turned down. Research suggests that voice constructiveness influences voice endorsement [7,11]. In sum, within the interactions among task groups, employees with a high sense of power will behave confidently and proactively when expressing suggestions and opinions. Supervisors will perceive these opinions as thoughtful and constructive, which leads to greater voice endorsement.

H2: Voice constructiveness mediates the relation between employees’ sense of power and voice endorsement.

### The moderating role of power distance and the moderated mediation effect

Power distance would moderate the effect of employees’ sense of power and their voice constructiveness. As an environmental characteristic that reflects employees’ participation in decision making, power distance reflects the extent to which individuals in groups or organizations expect and accept the inequality of power distribution [25]. In organizations with a high power distance, employees accept the convention that their duties are obeying and implementation, while decision making is a supervisor’s job. Thus, employees do not usually question the decisions that supervisors have made and tend to keep silent about job problems [26]. Moreover, employees who make suggestions are seen as trouble makers, and supervisors believe their opinions and advice are of low value [27]. Thus, in high power distance teams, no matter how strong an employee’s sense of power is, their supervisor would not take credit for their voice behavior, which leads to low perceptions of voice constructiveness for their advices. Thus, we propose the following hypothesis:

H3: Power distance negatively moderates the relationship between sense of power and voice constructiveness such that the relationship is weaker when power distance is higher.

Power distance would also moderate the indirect effect by which sense of power influences voice endorsement through the mediating role of voice constructiveness. Previous research has suggested the tendency of power distance in workplace would lead to less voice endorsement [28]. In work teams with a high power distance, there is a hidden rule that decisions are made by the supervisor and the employee’s job is to execute the decision according to the supervisor’s will. In this situation, a supervisor will perceive the employee’s suggestions as useless for improving organizational function and avoiding potential problems, and thus will reject their suggestions. In contrast, when a work team has a low power distance, the supervisor will believe in the improvement of organizational performance or the avoidance of work problems due to every team member’s effort, and will prioritize their opinions and suggestions, which will lead to greater voice endorsement. Thus, we propose the following hypothesis:

H4: Power distance moderates the indirect effect by which sense of power influences voice endorsement through the mediating role of voice constructiveness, such that the indirect effect is weaker when power distance is higher.

**Fig 1:**
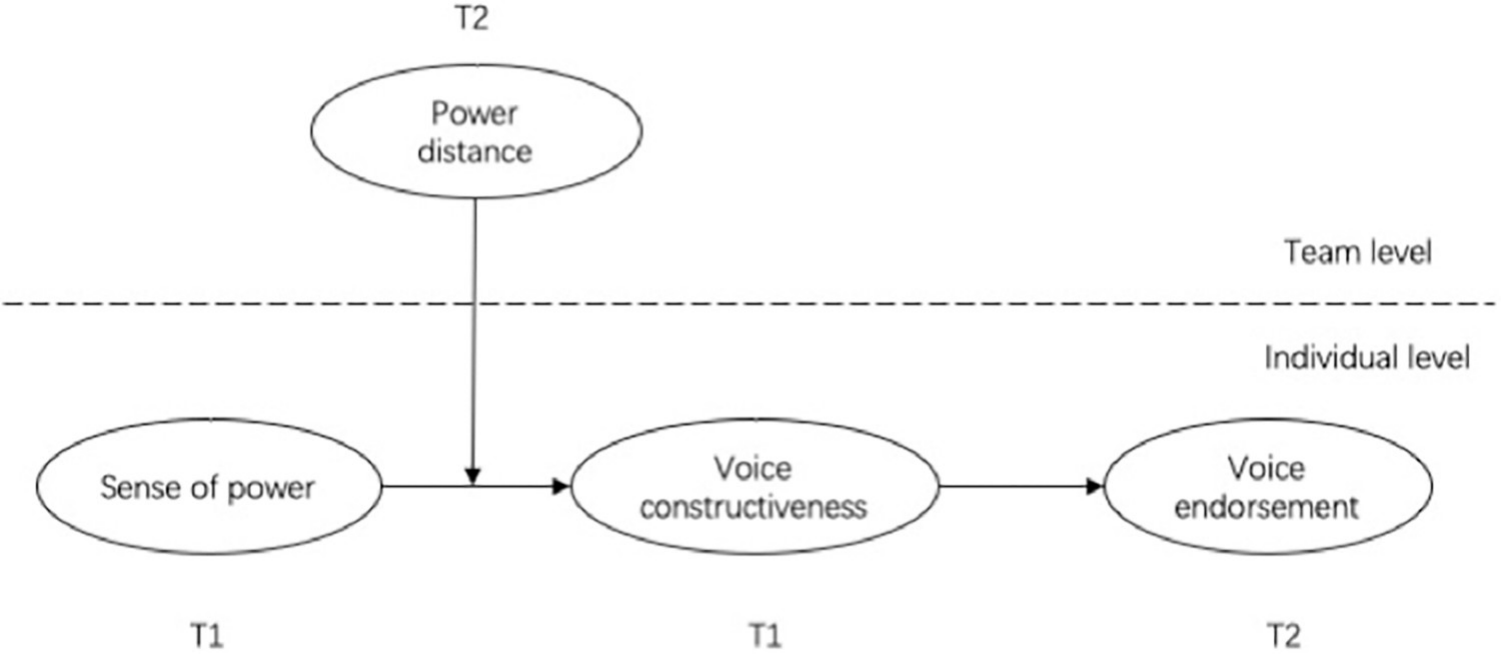
The theoretical model.

The theoretical model is shown in Fig 1.

## Materials and methods

### Sample and procedure

To collect data for our model, we used a two-time lagged design and paired questionnaire survey of supervisors and employees from an electric company in mainland China. At T1, employees reported their age, gender, education level, tenure, and sense of power, and supervisors reported the voice constructiveness of the employees. One month later, employees reported power distance and supervisors reported voice endorsement. This study was approved by the ethics review committee of Business School of NingboTech University (No. NBTBS2021002). Participants were informed that their responses were kept confidential and only be used for research purposes, and that they can discontinue their participation at any moment. At the end of the study, participants were debriefed and thanked. At T1, we sent out 363 questionnaires to 63 team groups, resulting in 321 responses from 61 team groups. At T2, the survey was completed by 307 of those 321 individuals, from 60 team groups. Each team consisted of four to six people. Among the sample, 147 participants (47.9%) were male and 160 (52.1%) were female; the average age was 34 years (SD = 9.20), the average tenure was 11.6 years (SD = 10.58), and 84.7% had a Bachelor’s degree or higher.

### Measurement

The measures used in this research were originally in English and were translated into Chinese using a standard translation and back-translation procedure [29]. Five-point Likert-type scales (1 = strongly disagree, 5 = strongly agree) were used for all measurements expect power distance, for which a seven-point scale (1 = strongly disagree, 7 = strongly agree) was applied.

#### Sense of power

This is an eight-item scale developed by these reserchers [23]. A sample item is “I can get him/her/them to listen to what I say” (α = .81).

#### Voice constructiveness

Two items from these reserchers [9], which originated from these reserchers [30], were used. A sample item is “This employee’s suggestions are likely to enhance the performance of his/her work group” (α = .74).

#### Voice endorsement

Three items from these reserchers [11], which were originally used by these reserchers [31], were used. A sample item is “I agree with this person’s comments” (α = .86).

#### Power distance

Six items from [25] were used. These items were developed by these reserchers [32] and have acceptable reliability [33]. A sample item is “Managers should make most decisions without consulting subordinates” (α = .89). In this study, we aimed to investigate whether the individual level variables of sense of power, voice constructiveness and voice endorsement change as a function of higher-order moderator variable of power distance in the workplace, thus, power distance is considered as a team-level variable. The average Rwg is 0.76, ICC(1) = .27, ICC(2) = .66, supporting aggregation at the team level [34].

#### Control variables

Based on previous theoretical research [9], we controlled employees’ demographic variables, including age, gender, education level, and tenure.

### Data analysis

Mplus 7.0 [35] was used to test the impact of sense of power on voice endorsement, and its indirect effect on supervisors’ voice endorsement through voice constructiveness. R was used to assess the Monte Carlo confidence intervals for indirect effect [36]. Mplus 7.0 was used to test the cross-level moderation effect of power distance and the moderated mediation effect, while the moderated mediation effect was examined using the Monte Carlo method. We used HLM to illustrate the moderation effect of power distance [37].

## Results

Confirmatory factor analysis revealed a good fit between the observed data and our hypothesized model (χ^2^ = 74.59, df = 38, CFI = .98, TLI = .97, RMSEA = .06, SRMR = .04), indicating satisfactory discriminant validity. The descriptive analysis (Table 1) shows that there is a significantly positive relation between sense of power and voice constructiveness (γ = .47, p< .01) and voice endorsement (γ = .33, p < .01), and between voice constructiveness and voice endorsement (γ = .38, p < .01), supporting our hypotheses.

**Table 1:** Descriptive statistics and correlations.

Variable	*M*	*SD*	1	2	3	4
1. Sense of power	3.44	.66	*(*.*81)*			
2. Voice constructiveness	3.96	.69	.47**	*(*.*74)*		
3. Voice endorsement	3.88	.71	.33**	.38**	*(*.*86)*	
4. Power distance	4.19	1.16	-.20**	-.08	-.01	*(*.*89)*

Note: *N* = 307.

* *p* < .05.

** *p* < .01 (two tailed).

### Main effect and mediation effect

Hypothesis 1 predicted that employees’ sense of power positively relates to voice endorsement. Our analysis (Table 2) revealed a significant direct effect of sense of power on voice constructiveness (β = .36, p < .01). Hypothesis 1 was thus supported. Hypothesis 2 predicted that voice constructiveness mediates the relation between sense of power and voice endorsement. The result shows that the indirect effect of sense of power on voice endorsement through voice constructiveness was significant (β = .15, 95% CI [.082, .214]). We used the Monte Carlo method to test the confidence intervals (CIs) of this indirect effect. A 95% CI for 20,000 simulated sampling did not include zero (CI [.086, .218]), so Hypothesis 2 was also supported.

**Table 2:** Direct effect and indirect effect analyses.

Variable	Model 1	Model 2	Model 3
	Voice endorsement	Voice constructiveness	Voice endorsement
**Direct effect**			
Sense of power	.36**	.49**	.21**
Voice constructiveness			.30**
**Indirect effect**			
Sense of power → Voice constructiveness →Voice endorsement			.15 [.082, .214]
**Monte Carlo method**			**95% CI, 20,000 sampling**
Sense of power → Voice constructiveness →Voice endorsement			[.086, .218]

Note: *N* = 307.

* *p* < .05.

** *p* < .01 (two tailed). The coefficients are non-standardized.

### Cross-level moderation effect and moderated mediation effect test

Hypothesis 3 predicted the moderation role of power distance in the relation between sense of power and voice constructiveness. Our results show that in the high power distance groups (1 SD higher than the average), the effect of sense of power and voice constructiveness was lower (γ = .37, CI [.460, .795]); for the groups with a low power distance (1 SD lower than the average), this correlation was higher (γ = .63, CI [.212, .523]), and the difference between the high power distance and low power distance groups was significant (γ = −.26, CI [−.518, −.002]). Thus, Hypothesis 3 was also supported.

Hypothesis 4 predicted that power distance moderates the indirect effect of sense of power on voice endorsement through voice constructiveness. The results show that this indirect effect was significant in the high power distance group (γ = .10, CI = [.038, .167]) and low power distance group (γ = .18, CI [.086, .268]) were both significant, and had significant differences (γ = −. 08, CI = [.150, .001]).

We used the Monte Carlo method to determine confidence intervals for these indirect effects. For the high power distance group, the confidence interval for the indirect effect of sense of power–voice constructiveness–voice endorsement did not include zero (CI [.060, .185]). This indirect effect was also significant for the low power distance groups (CI = [.086, .245]). Moreover, the difference between these indirect effects was significant (CI = [−.095, −.003]). See Table 3. Thus, Hypothesis 4 was also supported.

**Table 3:** Moderated mediation effect of team level power distance.

		Effects			Indirect effect
Dependent variable	Moderated variable Power distance	Effect 1 (*P*_*MX*_)	Effect 2 (*P*_*YM*_)	Indirect effect (*P*_MX * PYM_)	Monte Carlo method: 95% CI, 20,000 sampling
Voice endorsement	Low (−1 *SD*)	.37**	.28**	.18**	[.086, .245]
	High (+1 *SD*)	.63**		.10**	[.060, .185]
	Differences between high and low	−.27*		−.08	[−.095, −.003]

Note: *P*_*MX*_ is the effect of sense of power on voice constructiveness; *P*_*YM*_ is the effect of voice constructiveness on voice endorsement; *P*_*MX*_

_* PYM_ is the indirect effect of sense of power on voice endorsement.

## Discussion

Based on expectation status theory, we examined the underlying mechanism and boundary conditions of the effect of employees’ sense of power on supervisors’ voice endorsement. The results indicate that the voices of employees with a high sense of power will be endowed with higher constructiveness, which will lead to greater voice endorsement. When a work team has high power distance, supervisors will think that employees who raise questions about the current situation are going against work regulations; their voices will be considered less constructive, which will lead to less endorsement.

### Theoretical contributions and practical implications

We make several theoretical contributions and practical implications for research and management.

There are three theoretical contributions. First, by applying expectation status theory, this study enriches our knowledge about the antecedents of voice endorsement. Previous research has shown that the politeness of an employee’s voice [11], leader–member exchange [9,38], and the advisor’s characteristics [7] are among the key antecedents that influence voice endorsement. According to expectation status theory, an employee’s sense of power can trigger a more proactive work attitude in their work process and greater confidence in their voice behavior [12]. These behavioral cues will help supervisors build a higher performance expectation for employees with a high sense of power and their voice will be more easily endorsed. By introducing expectation status theory to the field of voice endorsement, we can discuss the relationship between individual influence and voice endorsement, which is a valuable addition to the theoretical research.

Second, by examining the mediating role of voice constructiveness, we reveal the mechanism underlying the influence of employees’ sense of power on voice endorsement. Our results indicate that voice endorsement not only relies on the content of the voice, but also on the characteristics of those who speak up. In Chinese culture, there is an idiom: “When a man is in a low position, his advice is of little effect.” Our results indicate that the reason for this “little effect” is that supervisors perceive the advice of people in low positions to be less constructive. Previous research has shown that perceived motivation, trust toward employees [7] and perceived threat [39] are key mechanisms explaining voice endorsement. In this study, we found that perception of voice constructiveness is an important factor in clarifying the tendency of voice endorsement, which supports the results of previous research [9] and enriches our understanding of the mechanism of voice endorsement in the Chinese context.

Third, by exploring the situational boundary mechanism of the effect of an employee’s sense of power on voice endorsement, we provide insights for management practice. Early research indicated that power distance inhibits employees’ voice behavior [25,40], but no research has focused on whether or how power distance influences voice endorsement from a supervisor’s perspective. Our results suggest that when a work team’s power distance is high, supervisors will believe employees’ duty is to executing the supervisor’s decisions and refrain from making suggestions about the current situation. Under this circumstance, employees’ voice behavior will be considered as creating trouble; thus, the constructiveness of their voice will be weakened, leading to lower voice endorsement.

The practical implications for management can be inspired by our findings that employee’s sense of power is positively related with supervisor’s voice endorsement. Based on the expectation status theory, employees with stronger sense of power would lead to higher perception of their voice constructiveness. As sense of power can be developed by controllable work content and workplace atmosphere, organizational practices can focus on increasing employees’ sense of power by endowing them more job autonomy [41], or creating a workplace climate where job crafting is encouraged [42]. Moreover, as power distance can reduce the association between employees’ sense of power and the supervisor’s perception of voice constructiveness, which further lead to less voice endorsement. Thus, supervisors in an organization could therefore make an effort to create a low power distance culture. Meanwhile, besides supporting employees to speak and make suggestions freely, organizations should also pay attention to the first-line managers’ perceptions of employees’ voice constructiveness, encouraging them to endorse the advice and suggestions of employees. This could be achieved by, for example, keeping the office door open during working hours, trying to avoid arranging separate seats for supervisors and employees, minimizing the differences in office conditions between supervisors and employees, and arranging more open means of communication, such as round-table meetings.

### Limitations and future research

This research has some limitations. First, our sample size was limited, which might have weakened the explanatory power of our conclusions [43]. Furthermore, there are other factors influencing voice endorsement, for example, leaders’ self-efficacy [44] and leader–member exchange [9]. However, our sample size was not sufficient to allow us to include those factors in the hypothesized model. Further research with a larger sample size would be valuable.

Second, the cross-sectional nature of the study means that we cannot draw conclusions about causal relationships among the variables. Researchers usually assume that a cross-sectional design is capable of providing correlational evidence, while experimental or quasi-experimental research can reveal the causal paths [45]. Thus, it is recommended that future studies with a quasi-experimental or experimental design are used to further investigate the relationship between employees’ sense of power and voice endorsement, for example by priming the sense of power before observing voice endorsement to enhance understanding of the causality of this relationship.

Third, the effect of social desirability might exist within self-reported data [46]. For example, supervisors might tend to endow high voice constructiveness to employees with whom they have high-quality leader–member exchange [9], but we did not control this variable in this research. Although we applied the two-time lagged design and paired questionnaire method to reduce the possibility of social desirability, more effort should be made to control other effects to enhance the reliability of the research results.

## Conclusion

Based on expectation status theory, we focused on explaining the relationship between employees’ sense of power and supervisors’ voice endorsement. By collecting data one month apart in a two-time lagged design with a paired questionnaire survey of 307 employees in 60 work teams, we found that (1) employees’ sense of power was positively related to supervisors’ voice endorsement; (2) supervisors’ voice constructiveness mediated the aforementioned relation; (3) power distance at the team level negatively moderated the influence of employees’ sense of power on supervisors’ voice constructiveness; and (4) power distance at the team level also negatively moderated the indirect effect of sense of power on voice endorsement.

## Supporting information

S1 File (DOCX)
